# The Influence of Nesfatin-1 on Bone Metabolism Markers Concentration, Densitometric, Tomographic and Mechanical Parameters of Skeletal System of Rats in the Conditions of Established Osteopenia

**DOI:** 10.3390/ani12050654

**Published:** 2022-03-04

**Authors:** Grzegorz Tymicki, Iwona Puzio, Marta Pawłowska-Olszewska, Marek Bieńko, Radosław Piotr Radzki

**Affiliations:** Department of Animal Physiology, Faculty of Veterinary Medicine, University of Life Sciences in Lublin, Akademicka St. 12, 20-950 Lublin, Poland; g.tymicki@wp.pl (G.T.); martapaw@autograf.pl (M.P.-O.); marek.bienko@up.lublin.pl (M.B.); radoslaw.radzki@up.lublin.pl (R.P.R.)

**Keywords:** nesfatin-1, osteopenia, rat, pQCT, DXA, bone strength

## Abstract

**Simple Summary:**

Nesfatin-1 is an adipokine with little known effect on the skeletal system. In this study, we examined the effect of 8-wk administration of nesfatin-1 on densitometric, tomographic, and mechanical parameters of bones, as well as the concentration of bone metabolism markers in rats with experimentally induced established osteopenia.

**Abstract:**

Our study aimed to evaluate the impact of nesfatin-1 administration on bone metabolism and properties in established osteopenia in ovariectomized female rats. In total, 21 female Wistar rats were assigned to two groups: sham-operated (SHAM, *n* = 7) and ovariectomized (OVA, *n* = 14). After 12 weeks of osteopenia induction in the OVA females, the animals were given i.p. physiological saline (OVA, *n* = 7) or 2 µg/kg body weight of nesfatin-1(NES, *n* = 7) for the next 8 weeks. The SHAM animals received physiological saline at the same time. Final body weight, total bone mineral density and content of the skeleton were estimated. Then, isolated femora and tibias were subjected to densitometric, tomographic, and mechanical tests. Bone metabolism markers, i.e., osteocalcin, bone specific alkaline phosphatase (bALP), and crosslinked N-terminal telopeptide of type I collagen (NTx) were determined in serum using an ELISA kit. Ovariectomy led to negative changes in bone metabolism associated with increased resorption, thus diminishing the densitometric, tomographic, and mechanical parameters. In turn, the administration of nesfatin-1 led to an increase in the value of the majority of the tested parameters of bones. The lowest bALP concentration and the highest NTx concentration were found in the OVA females. The bALP concentration was significantly higher after nesfatin-1 administration in comparison to the OVA rats. In conclusion, the results indicate that nesfatin-1 treatment limits bone loss, preserves bone architecture, and increases bone strength in condition of established osteopenia.

## 1. Introduction

The skeleton is a dynamic, metabolically active structure that ensures movement, protection of internal organs, and maintenance of the mineral balance. In addition, bone is the target tissue for various biologically active substances that may affect its size, metabolism, and, consequently, its histological structure and strength. Bone health and strength are very important to the lifestyle of animals. They ensure the locomotion, the ability to escape, food and water access, and copulation activity. Unfortunately, lameness, leg abnormalities, and bone fractures are common in domestic and companion animals. These disorders can be associated with genetics, anatomical traits, nutrition, gut health, infectious diseases, lighting programs, movement frequency, and toxicities [1,2,3,4,5,6,7,8,9,10,11,12]. Bone disorders occurring in animals are, e.g., rickets, osteomalacia, osteochondrosis, osteoarthritis, dyschondroplasia, osteoporosis. [1,3,6,8,13,14,15,16]. Furthermore, bone disorders in animals are diagnosed quite late when clinical signs are present. Therefore, it is important to study the role of various factors in bone metabolism and their possible application in the treatment of animals.

In recent years, many new biologically active peptides have been identified. Some of them are adipokines released from adipose tissue. According to recent studies, adipokines are involved in bone metabolism, but their role is not fully understood [17,18,19,20,21,22,23,24,25]. One of the adipokines with little known effect on the skeleton is nesfatin-1 [26]. Nesfatin-1 was described by Oh-I and co-workers in 2006 as an anorexigenic 82-amino acid peptide [27]. Nesfatin-1 (AA 1-82) and other peptides, nesfatin-2 (AA 85-163) and nesfatin-3 (AA166-396), are formed from their precursor, nucleobindin-2 (NUCB2), produced by bone cells [28]. Previous studies indicated that only nesfatin-1 exerts a biological effect [27,29,30]. The central and peripheral location of nesfatin-1 has been determined. The first studies demonstrated the expression of *NUCB2* mRNA and NUCB2/nesfatin-1 protein in areas of the rodent hypothalamus and brainstem related to the regulation of food intake [27,29]. Then, its location was affirmed in other areas of the hypothalamus, midbrain, hindbrain, sympathetic and parasympathetic spinal cord neurons [31,32,33,34,35,36]. Peripheral nesfatin-1 has been detected in the gastrointestinal tract, heart, lung, reproductive system, adipocytes, joint cartilage, and growth cartilage [37,38,39,40,41,42,43,44,45]. The presence of nesfatin-1 has also been demonstrated in synovial fluid and breast milk [40,46]. The studies have shown that nesfatin-1 exhibits pleiotropic activity, however in the databases there are only a few studies documenting the connection between nesfatin-1 and the skeletal system. On the one hand, the results of in vitro and in vivo studies indicated that nesfatin-1 is associated with development of osteoarthritis (OA) [40,47,48]. On the other hand, nesfatin-1 stimulated bone mineralization and inhibited osteoclastogenesis in an in vitro study [49]. The data on the influence of nesfatin-1 on bone in vivo are very limited [42,49]. Initially, Li and co-workers [49] demonstrated a positive changes in densitometric bone parameters after its intravenous application. In turn, a protective effect of intraperitoneal nesfatin-1 treatment on the densitometric, tomographic, and mechanical bone parameters in developing osteopenia in female rats was shown in our earlier study [42]. Unfortunately, the studies on its influence on skeletal system are sparse and need confirmation. In order to confirm whether nesfatin-1 does exert really a positive effect on bone we intended to examine its effect in established osteopenia induced by ovariectomy in rats. The effect of nesfatin-1 on bone tissue was studied based on the evaluation of densitomeric, tomographic and mechanical parameters of bones, as well as the concentration of bone metabolism markers.

## 2. Materials and Methods

### 2.1. Animal Procedures

The research was accepted by the 2nd Local Ethics Committee for Animal Experiments in Lublin, Poland (approval no. 23/2015).

Three-month-old healthy female Wistar rats (*n* = 21) were used in study. The initial body weight (BW) of rats was approximately 210–230 g. During the experiment, the rats were kept in experimental plastic cages (2–3 rats) in controlled conditions at 22 ± 2 °C, with the 12/12 h day/night ratio and with lab chow (LSM, Agropol, Poland) and water available ad libitum. The access to food was limited only one night before surgery and euthanasia. After 7-d acclimatization, the rats were randomly assigned to 2 groups intended for pseudogonadectomy surgery (SHAM, *n* = 7) and ovariectomy (OVA, *n* = 14) according to the procedure described previously [42]. The general anesthesia was applied during the surgical operations (i.m. injection of 0.05 mg/kg BW atropinum sulphuricum, Polfa-Warszawa, Poland; 3 mg/kg BW ketamine and 10 mg/kg BW xylazine, Biowet-Pulawy, Poland). After the surgery, the rats were maintained individually for several days. No postoperative complications under veterinary examinations were observed. Postoperative analgesia was not used.

In the next step of the experiment, the animals (after pseudo-gonadectomy surgery and ovariectomy) were housed in the vivarium for 12-wks for induction of osteopenia in the ovariectomized rats. After this period, the ovariectomized rats were randomly allocated into 2 groups receiving daily by i.p. injection physiological saline (PhS) (OVA, *n* = 7) or 2 μg/kg BW of nesfatin-1 (Phoenix Pharmaceuticals, Inc., Burlingame, CA, USA) (NES, *n* = 7) for 8-wks. The SHAM animals received PhS at the same time. Nesfatin-1 was diluted in PhS (0.2 µg nesfatin-1/0.1 mL PhS). The volumes of the administered solution of nesfatin-1 and PhS were corresponding. In order to eliminate the effect of nesfatin-1 on food intake, the injections were made in the morning hours up to 12 pm. During the experiment, BW of rats was assessed every two days to calculate administered nesfatin-1. Moreover, the feed intake was monitored.

At the end of an 8-wk experimental administration of PhS and nesfatin -1, the animals were weighed and then euthanized by CO_2_ overdose. After blood collection via intraventricular puncture, euthanasia was confirmed by cervical dislocation. Subsequently, the total skeletons were examined through dual-energy X-ray absorptiometry (DXA). Right femora and tibias were isolated, and bones cleaned of soft tissues were weighed, measured, and stored at −20 °C for further assays.

### 2.2. Bone Densitometry Measurements

**Figure 1:**
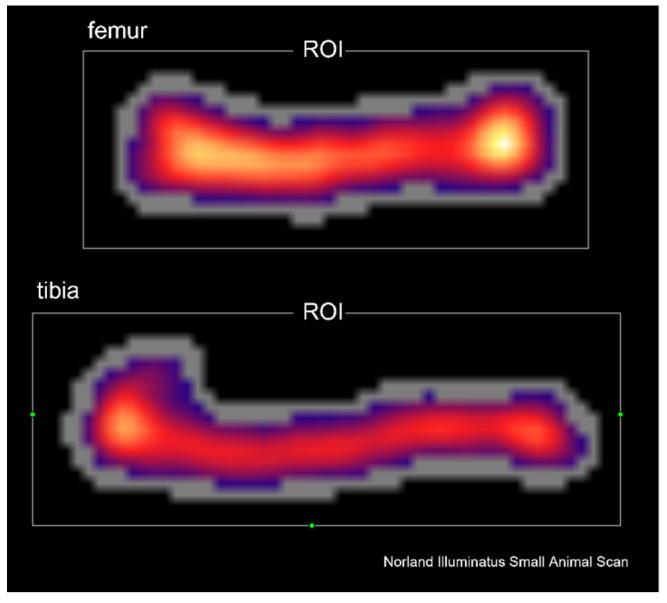
Representative DXA scan of femur and tibia. Abbreviations: ROI—region of interest.

The Norland Excel Plus device (Fort Atkinson, WI, USA) and the software supplied by the manufacturer (Small Subject Scan 4.4.1) were used for DXA analysis of the total skeleton mineral content (totBMC) and mineral density (totBMD), as well as mineral content (BMC) and mineral density (BMD) of femora and tibias (Figure 1). The prescan were made at the speed of 100 mm/s and resolution of 1.5 × 1.5 mm. The scan parameters were 30 mm/s speed and 1.0 × 1.0 mm resolution. The calibration of devoice was performed before each measurement cycle.

### 2.3. Bone Tomographic Measurements

**Figure 2:**
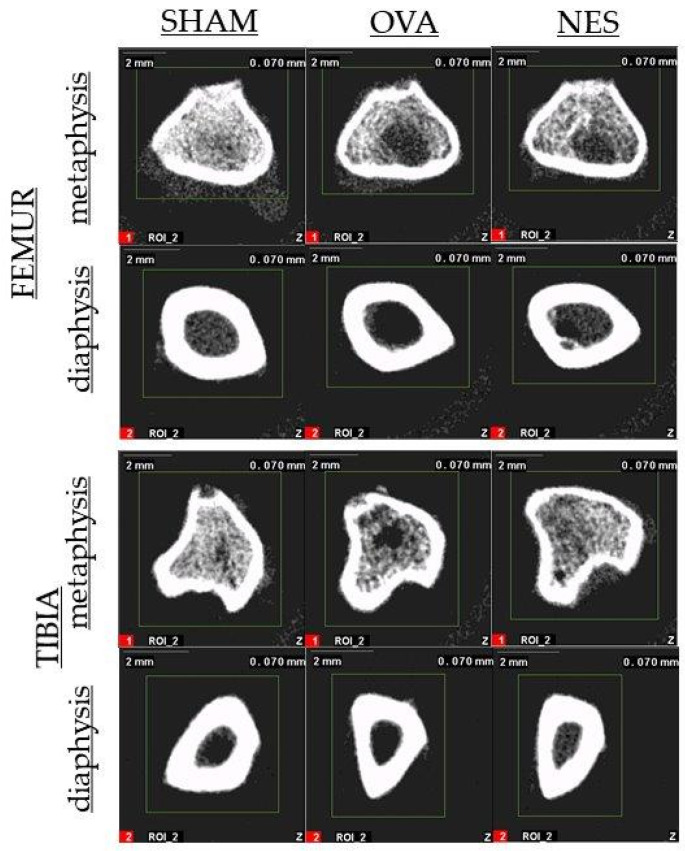
Representative pQCT scan of metaphysis and diaphysis of femur and tibia.

Femora and tibias were examined by a peripheral quantitative computed tomography (pQCT) using XCT Research SA Plus and software v.6.2.C (Stratec Medizintechnik, GmbH, Pforzheim, Germany). The trabecular bone tissue was scanned in the proximal metaphysis of the tibia and in the distal metaphysis of the femur (Figure 2) while cortical bone tissue was analyzed in the middle of the diaphysis length. The threshold was determined to be 0.630 cm^−1^ for trabecular bone and 0.790 cm^−1^ for cortical bone. The speed was set at 10 mm/s for the initial scan and at 4 mm/min for the measurement scan. The apparatus was calibrated before every measurement session using a quality control phantom. The tomographic parameters were determined at 50% length of the diaphysis and in the metaphyses as previously described [42].

### 2.4. Bone Mechanical Analysis

Three-point bending Zwick Z010 testing system (Zwick-146 Roell GmbH & Co., Ulm, Germany) supported by testXpert II 3.1 software was used to determine bone strength. After placing the bone on 2 supports (40% of the mean length of the bone), the moving head (1 kN, Xforce HP series) was operated perpendicularly at a constant speed (10 mm/min) in the middle of the diaphysis [42,50]. Based on the load–displacement curve the ultimate strength, Young’s modulus, and work to fracture were estimated as bone strength parameters.

### 2.5. Bone Markers Analysis

Serum concentrations of bone specific alkaline phosphatase (bALP), osteocalcin, and crosslinked N-terminal telopeptide of type I collagen (NTx) were measured using enzyme-linked immunosorbent assay (ELISA) and microplate reader (Bio-Rad Laboratories Inc., Hercules, LA, USA). The commercial rat-specific kits were used in the tests (Sunred Biotechnology Company, Shanghai, China) according to the manufacturer’s protocols. Serum content of calcium and phosphorus was determined spectrophotometrically using the respective commercial kits (Alphadiagnostic, Warsaw, Poland) and biochemical analyzer Mindray BS-120 (Shenzhen Mindray Bio-Medical Electronics Co., Ltd., Shenzhen, China).

### 2.6. Statistical Analysis

The values of all the parameters are reported as mean values together with S.E.M. The one-way analysis of variance (ANOVA) and the Tukey post hoc test were used to determine differences between the means. The means were exhibited statistically significant at *p* ≤ 0.05. Data were analyzed using STATISTICA version 13.1 (StatSoft, Inc., Tulsa, OK, USA).

## 3. Results

### 3.1. Body Weight, Feed Intake, Bone Mass, and Length

There were no significant differences in the BW of the rats during the treatment period (data now shown). Moreover, the assessment of rats’ BW at the end of the experiment did not show statistically significant differences between the individual groups (SHAM—284.6 g ± 10.15, OVA—309.3 g ± 21.23, NES—278 1 g ± 5.69). However, the highest BW values were found in the OVA rats. We did not observe a differences between studied groups in feed intake (results not shown). The SHAM rats were characterized by the greatest values of femur and tibia mass, compared to both ovariectomized groups (Table 1). Similarly, the measurements of the femur length showed the highest values of this parameter in the SHAM animals (Table 1).

### 3.2. Bone Densitometry Measurements

The OVA animals were characterized by statistically significant lower totBMD values in relation to the SHAM and NES females. The totBMD in the SHAM and NES females was similar (Table 1). A similar trend was found for totBMC, but the differences between the particular groups did not show statistical significance (Table 1). The ovariectomy significantly reduced the BMD and BMC of both bones, compared to the SHAM females. The values of femur BMC, as well as tibia BMD and BMC in the NES rats were significantly greater than in the OVA rats and similar to the values in the SHAM rats (Table 1).

### 3.3. pQCT Analysis of Isolated Bones

The highest values of trabecular bone parameters, i.e., T.BMC, Tv.BMD, Tb.BMC, and Tb.BMD of the distal metaphysis of the femur were stated in the SHAM rats (Table 2), whilst the lowest values of these parameters were recorded in the OVA group. The significantly higher values of T.BMC, Totv.BMD, Tb.BMC, Tb.BMD were observed in the NES rats in relation to the OVA rats. There were no statistically significant differences in T.A and Tb.A between the groups (Table 2). Femur diaphysis of OVA rats characterized by the lowest values of T.BMD (Table 2). However, the values of this parameter in the SHAM and NES animals were similar. The results of the other tomografic parameters of femur diaphysis (T.BMC, Ctr.BMC, T.A, Ctr.A, Ctr.Th, Peri.C, Endo.C, and SSI) were similar in all groups (Table 2).

Significantly reduced T.BMC, Tv.BMD, Tb.BMD, and Tb.BMC values of the tibia metaphysis, in comparison to those in the group of the SHAM females, were found in the OVA females (Table 3). The NES females were characterized by significantly higher Tv.BMD and Tb.BMD values than those recorded for the OVA females. The values of T.A and Tb.A were at similar level in all examined groups (Table 3). The tibia diaphysis in the SHAM rats had significantly higher values of T.BMC, T.A, Ctr.A, Peri.C, and SSI compared to the OVA and NES groups. The Ctr.BMD and Ctr.BMC of the tibias were also higher in the SHAM rats than in the OVA rats (*p* ≤ 0.05). The values of these parameters were similar in SHAM and NES groups. The mean values of the tibia T.BMD, Ctr.Th, and Endo.C did not show statistically significant differences between the study groups (Table 3).

### 3.4. Mechanical Parameters of Isolated Bones

The 3-point bending test indicated a significant decrease in the values of femur ultimate strength and the work to fracture in OVA group (Table 4). The values of the abovementioned parameters in the SHAM and NES animals were similar. No significance differences were observed for femur Young’s modulus between the study groups. The tibias of the NES females showed higher values of Young’s modulus than the values noted in the SHAM females. The mean values of tibia ultimate strength and work to fracture did not differ significantly between the groups, although the lowest values were found in the OVA group (Table 4).

### 3.5. Analysis of Bone Metabolism Markers

The OVA animals were characterized by the lowest bALP concentration and the highest NTx concentration (Table 5). The concentration of bALP and NTx in the SHAM and NES groups were similar. The bALP concentration significantly increased in the NES group in comparison to the OVA group. No significant differences were stated in the osteocalcin level (Table 5). The concentration of serum ionized calcium and phosphorus was similar in the examined groups (Table 5).

## 4. Discussion

The present study was conducted to examine the influence of nesfatin-1 on bone metabolism and properties in established osteopenia as a consequence of bilateral ovariectomy in female rats. This experimental procedure was intended to imitate the internal environment of deficiency of sex hormones and causing osteopenia/osteoporosis in experimental animals [51]. The decrease in estrogen levels that progresses with age or results from gonadectomy in mature individuals increases bone metabolism. The loss of ovarian function causes an imbalance between bone resorption and formation, which, in turn, reduces the bone mass [52,53,54]. Moreover, the increased bone turnover negatively influences bone microarchitecture leading to a reduction in bone strength to load and an increased risk of fracture [55,56]. Bone strength is determined by, e.g., the size, geometry, cortical bone porosity, trabecular bone morphology, and mineral density [57,58,59,60]. In vivo, it is indirectly estimated primarily based on BMD and BMC determined with the DXA method. In animals, the use of the DXA method in combination with special software designed for testing small animals facilitates assessment of totBMD and totBMC, as well as BMD and BMC for individual skeletal fragments. It has been proven that strength of bone is strongly associated with BMD, and the risk of fracture increases in individuals with low values of this parameter [61,62,63].

In our study, the experimental ovariectomy caused a significant reduction in femur and tibia bone mass, totBMD, BMD, and BMC values. Moreover, reduced values of the pQCT and strength parameters were observed. These results indicate predominance of bone resorption over bone synthesis, and, consequently, a reduction in bone mineralization. In turn, in the 12-week absence of the action of sex hormones, nesfatin-1 was found to mitigate the negative changes in densitometric parameters. In the ovariectomized rats receiving nesfatin-1, the BMD and BMC values were higher by 8.9% and 12.8% in the femur and by 5.4% and 9.6% in the tibia, compared to the values noted in the PhS-administered OVA rats. In addition, higher totBMD values (by 6.25%) were found in NES animals, compared to OVA. Moreover, the values of these parameters were similar to those found in the group of females with preserved gonadal function. These results confirmed our earlier observation in a study performed in ovariectomized rats with developing osteopenia, where 8-wk i.p. administration of nesfatin-1 mitigated adverse changes in DXA parameters of tibia and femur [42]. Similarly, intravenous administration of nesfatin-1 limited the reduction in BMD values in the study of Li et al. [49]. These authors observed an augmentation in BMD values by 5% and 10% for femur and lumbar vertebrae by 10% and 5%, respectively [49].

The reduction in the negative changes in bone tissue after i.p. administration of nesfatin-1 was confirmed by its positive effect on the tested tomographic parameters, compared to the OVA females. The mean values of the distal metaphysis of the femur were significantly increased after the nesfatin-1 administration, i.e., by 9.65% and 2.8% for T.BMC and Tv.BMD and by 31.6% and 20.5% for Tb.BMC and Tb.BMD, respectively. In the case of the tibia, the positive effects of nesfatin-1 were also manifested as the 8.75% and 56% increase in the Tv.BMD and Tb.BMD values, respectively, compared to the OVA group. In the case of cortical bone tissue, a significant effect of nesfatin-1 was only observed in relation to the femur T.BMD (an increase by 2.56% vs. OVA) and the tibia Ctr.BMD (an increase by 5.19% vs. OVA). This study and our previous investigations in rats with developing osteopenia [42] prove that nesfatin-1 has s a positive effect mainly on trabecular bone pQCT parameters determined in metaphyses.

In addition, the limitation of the negative changes in bone tissue as result of effects of sex hormone deficiency by nesfatin-1 administration to the gonadectomized rats was evidenced by the enhancement in the mechanical strength. The mean value of ultimate strength and the work-to-fracture of the femur in the NES group, in comparison with the OVA group receiving PhS, increased by 28.48% and 29.82% and these value were closer to the results obtained in the animals with preserved gonadal function. In the case of the tibia, the values of ultimate strength and the work to fracture in the NES animals were higher by 16.24% and 14.38% than in the OVA animals; however, these differences were not statistically confirmed.

In the available literature, there are papers presenting the influence of other adipokines on bone mass, DXA and tomographic parameters, and bone strength. Unfortunately, they are not explicit. Luo et al. [20] and Oshima et al. [21] reported a stimulating effect of adiponectin on osteoblasts (OB) proliferation and differentiation, and bone mineralization. Moreover, the enhancement of trabecular bone mass, the decreased number of osteoclasts (OC), and the diminished NTx concentration suggest a positive effect of adiponectin on bone formation and bone mass [21]. On the other hand, negative association of adiponectin with bone mass and strength was observed in mice [18]. According to Wang et al. [64], adiponectin deficiency was found to protect against reduction in BMD and strength parameters in ovariectomized mice. However, Haugen et al. [65] reported a positive correlation between adiponectin expression and ultimate bending moment and ultimate energy absorption, and a negative correlation with bending stiffness. Moreover, adiponectin enhanced leptin, collagen, and tumor necrosis factor-alpha (TNF-α) expression in OB, and stimulated OC proliferation without the influence on their functions [65].

Bone cell activity and bone metabolism can be assessed based on biochemical parameters of blood or urine, in which the concentration of so-called biochemical bone metabolism markers can be determined. These are regarded as diagnostic indicators for evaluation of bone turnover, which consists of bone formation and resorption [66]. The determination of such bone-specific proteins as osteocalcin synthesized by OB or enzymes secreted by these cells, e.g., bALP, is commonly used to assess the OB activity. Osteocalcin is the primary non-collagen protein in the bone matrix and a marker of osteosynthesis [67]. However, an increase in its concentration in the blood also indicates an increase in bone turnover [68]. In turn, the NTx concentration was used for assessment of OC activity and bone resorption [66]. In our studies, the changes in the bone tissue parameters were also accompanied by changes in the concentration of of NTx and bALP. The higher level of the NTx concentration in the OVA group, in comparison with the SHAM animals, indicates stimulation of bone resorption as a consequence of the ovariectomy and no impact of estrogens on the bones tissue. In turn, the rise in the bALP concentration after nesfatin-1 administration is evidence of an increase in OB activity. The consequence of change in OB activity is the intensification of osteosynthesis confirmed in the DXA, pQCT, and mechanical tests. The simultaneous reduction in the NTx concentration by 5.4% (vs. OVA) in the NES females may indicate that nesfatin-1 inhibits bone resorption caused by the decrease in the estrogen level. These results are in line with our previous observations [42] and confirm that nesftatin-1 participates in the metabolism of bone tissue, mediating in both osteosynthesis and resorption. However, in the study in rats with developing osteopenia, we also observed increasing concentrations of osteocalcin after nesfatin-1 administration. In present study, no significant changes in this parameter were stated.

The osteoprotective action of estrogens on bone tissue proceeds in several ways. Estrogens affect OC activity via regulation of the relation between the receptor activator of nuclear factor kappa B ligand and osteoprotegerin (RANKL/OPG). RANKL concentration rises with decreasing levels of estrogens, which increases osteoclastogenesis and bone resorption. The osteoprotective effect of nesfatin-1 on bone tissue in estrogen deficiency also seems to be related to RANKL, as demonstrated Li et al. in vitro studies [49]. In their research, the influence of RANKL and nesfatin-1 on the activity of RAW 264.7 murine macrophage cells, OC precursor cells, was evaluated based on measurements of the tartrate-resistant acid phosphatase (TRAP) concentration, which is considered the primary marker of OC activity. It was found that nesfatin-1 inhibited the formation of polymorphic TRAP-positive cells and decreased TRAP activity. Thus, it is highly probable that the positive effect of nesfatin-1 on bone tissue parameters observed in present study is the result of its positive effect related to the inhibition of OC activity, which was confirmed by measurements of the NTx serum concentration.

The effects on OC activity and osteoclastogenesis have also been found in relation to other adipokines. Vaspin shows an inhibitory action on RANKL-stimulated osteoclastogenesis in vitro [19]. Chemerin deficiency inhibits differentiation of the osteoclast line [23]. Resistin induces OC formation and NF-kB promoter activity, and the expression of resistin increases with the degree of cell differentiation towards OC [24]. In turn, the effect of visfatin and adiponectin is unambiguous. On the one hand, visfatin acts as a suppressant; on the other hand, its deficiency inhibits the formation of OC [22,69]. Moreover, by induction of pro-inflammatory factors, i.e., interleukin-6 (IL-6), interleukin-8 (IL-8), and MCP-1 (monocyte chemotactic protein) in osteoblastic line cells, visfatin may indirectly influence OC formation [25,70]. Adiponectin has also been shown to inhibit as well as stimulate OC [20,21,71,72,73]. Nevertheless, despite the increased OC differentiation, no changes in OC activity under the influence of adiponectin were found either [65,73]. Adiponectin indirectly activates OC by stimulation of RANKL synthesis and inhibition of osteoprotegerin synthesis by OB [71,74]. Osteoprotegerin as a decoy receptor for RANKL inhibits of osteoclastogenesis and OC activity. Adiponectin can also indirectly influence OC, stimulating the synthesis of pro-inflammatory mediators, i.e., matrix metalloproteinase-1, matrix metalloproteinase-13, interleukin-1, interleukin-6, interleukin-8, and monocyte chemoattractant protein-1 by OB and osteoblastic line cells [75,76]. In addttion, nesfatin-1 stimulates pro-inflammatory mediators, i.e., cyclooxygenase-2, interleukin-6, interleukin-8, and macrophage inflammatory protein-1α in osteoarthritis primary chondrocytes [48]. Based on the abovementioned data, it can be assumed that nesfatin-1, together with other adipokines, is involved in the development of pathological changes in cartilage in which pro-inflammatory factors play an important role. Rather, these results suggest a negative impact of nesfatin-1 on the skeletal system, as pro-inflammatory cytokines participate in bone turnover and the pathogenesis of osteoporosis. They increase the activity of OC and bone resorption, leading to unfavorable changes in its structure and properties [77,78,79,80]. Taking the above into consideration, the induction of pro-inflammatory factors by nesfatin-1 may suggest its involvement in periarticular bone remodeling accompanying osteoarthritis on the one hand. In turn, considering the increased level of nesfatin-1 in blood and synovial fluid in individuals with osteoarthritis and the increase in its synthesis in vitro induced by proinflammatory cytokines, it may be assumed that this adipokine has a protective action.

However, the positive effect of nesfatin-1 on OB manifested in the increased formation of nodules during the mineralization process was found earlier an in vitro study [49]. Alkaline phosphatase (ALP) is involved in the mineralization process, and increased activity of ALP may indicate an increase in OB activity. However, Li et al. [49] presented a rise in ALP activity in preosteoblastic cells in an in vitro study only depending on recombinant bone morphogenetic protein -2, as nesfatin-1 alone did not cause an increase in OB activity. In the present study, we found increased serum bALP level indicating an increase in the activity of OB. This increase, combined with the improvement of tested DXA, pQCT, and strength parameters, confirms the positive influence of nesfatin-1 on bone tissue under conditions of established osteopenia.

It was found that other adipokines exerted effects on OB. Adiponectin acts directly on OB through Adipo1 and Adipo2 receptors [81]. It stimulates osteoblastogenesis by increasing the proliferation and differentiation of osteoblastic line cells and mineralization, while the OB express adiponectin [21,73,81]. The interaction of adiponectin is mediated by mitogen-activated kinases and bone morphogenetic protein-2 influencing OB differentiation [20,82,83]. Murine preosteoblasts and mature OB expressed resistin, which enhanced the proliferation of preosteoblasts in a protein kinase A (PKA)- and protein kinase C (PKC)-dependent manner [24]. In primary osteoarthritis OB, resistin increased Wnt signaling activation, OB metabolic activity, and bone formation [84]. An increase in OB proliferation, collagen I synthesis, and glucose uptake, was observed in vitro under the influence of visfatin [85]. Its expression increased with the degree of OB differentiation and was associated with a higher level of nicotinamide adenine dinucleotide (NAD) [86]. Lack of visfatin or inhibition of its effect reduces the level of NAD and osteogenesis, which suggests that the differentiation of osteoblastic line cells depends on the level of NAD, and visfatin may have a regulatory action in these processes [86]. The positive effect of visfatin on bone tissue was confirmed in studies conducted by Briana et al. [87] concerning the fetal and neonatal period. Lipocalin-2 (LCN-2, syderocalin,) is synthesized by OB and chondrocytes, and the level of its expression increases with the increasing degree of cell differentiation [17,88]. Transgenic mice with LCN-2 overexpression showed, however, unfavorable changes in growth cartilage, reduction in the osteosynthesis rate, intensification of bone resorption, and consequent bone mass reduction and changes in its microarchitecture—mainly reduction in bone trabeculae [17]. Moreover, chemerin found in preosteoblastic cells is likely to participate in OB differentiation [89].

Taking the above into account, the results regarding the influence of different adipokines on skeletal tissue are not conclusive and, sometimes, even contradictory. Moreover, their action takes place through various signaling pathways. Similarly, in the case of nesfatin-1, it is not yet possible to elucidate the precise mechanisms of its effects on bone and cartilage. On the one hand, the induction of pro-inflammatory cytokines in chondrocytes, which mediate in osteoclastogenesis, as well as the absence of changes in OB activity after in vitro treatment and, on the other hand, the inhibition of osteoclastogenesis in vitro and the positive effect on bone parameters in vivo necessitate further research. However, the results of DXA, pQCT and strength measurements of bones, and bone metabolism markers in the female rats with established osteopenia are undeniable.

## 5. Conclusions

Nesfatin-1 treatment limits bone loss, protects against architectural changes, and increases bone strength. The confirmed reduction in the osteopenic changes in established osteopenia suggests that exogenous nesfatin-1 can be used to treat osteopenic disorders of skeletal system. However, further research is still needed in this area.

## Figures and Tables

**Table 1:** DXA parameters of whole skeleton and isolated femur and tibia of female rats after the establishment of osteopenia.

Parameters	SHAM	OVA	NES	*p*-Value
totBMD	0.155 ± 0.002 ^a^	0.144 ± 0.001 ^b^	0.153 ± 0.002 ^a^	0.003
totBMC	9.56 ± 0.024	8.93 ± 0.176	9.53 ± 0.331	0.192
Femur				
Mass (g)	0.81 ± 0.008 ^a^	0.69 ± 0.013 ^b^	0.73 ± 0.029 ^b^	0.002
Length (mm)	35.6 ± 0.35 ^a^	33.7 ± 0.17 ^b^	34.0 ± 0.31 ^ab^	0.005
BMD (g/mm^2^)	0.132 ± 0.003 ^a^	0.112 ± 0.002 ^b^	0.122 ± 0.002 ^ab^	<0.001
BMC (g)	0.432 ± 0.013 ^a^	0.344 ± 0.007 ^b^	0.388 ± 0.014 ^a^	<0.001
Tibia				
Mass (g)	0.59 ± 0.016 ^a^	0.52 ± 0.108 ^b^	0.53 ± 0.023 ^b^	0.012
Length (mm)	38.99 ± 0.439	37.92 ± 0.159	38.44 ± 0.364	0.112
BMD (g/mm^2^)	0.103 ± 0.002 ^a^	0.089 ± 0.002 ^b^	0.095 ± 0.002 ^a^	0.002
BMC (g)	0.304 ± 0.011 ^a^	0.250 ± 0.009 ^b^	0.274 ± 0.007 ^a^	0.003

Data are expressed as means ± S.E.M. (*n* = 7 in each group); ^a,b^—means within a row with unlike superscripts differ significantly. Statistically significant differences were established at *p* ≤ 0.05. Abbreviations: totBMD—bone mineral density of the skeleton; totBMC—bone mineral content of the skeleton; BMD—bone mineral density; BMC—bone mineral content.

**Table 2:** Femur tomographic parameters of female rats after the establishment of osteopenia.

Parameters	SHAM	OVA	NES	*p*-Value
Distal metaphysis				
T.BMC (mg/mm)	15.2 ± 0.549 ^a^	11.4 ± 0.211 ^c^	12.5 ± 0.507 ^b^	<0.001
Tv.BMD (mg/mm^3^)	808 ± 16.84 ^a^	652 ± 11.17 ^c^	670 ± 17.57 ^b^	<0.001
Tb.BMC (mg/mm)	4.5 ± 0.234 ^a^	1.9 ± 0.179 ^c^	2.5 ± 0.218 ^b^	<0.001
Tb.BMD (mg/mm^3^)	530 ± 22.46 ^a^	242 ± 19.55 ^c^	292 ± 21.36 ^b^	<0.001
T.A (mm^2^)	19.0 ± 0.462	17.5 ± 0.422	18.6 ± 0.775	0.182
Tb.A (mm^2^)	8.6 ± 0.205	7.9 ± 0.187	8.4 ± 0.35	0.181
Diaphysis				
T.BMC (mg/mm)	9.9 ± 0.191	9.0 ± 0.206	9.3 ± 0.262	0.057
T.BMD (mg/mm^3^)	1039 ± 14.64 ^a^	977 ± 13.29 ^b^	1002 ± 13.47 ^a^	0.009
Ctr.BMC (mg/mm)	9.0 ± 0.233	8.4 ± 0.213	8.7±0.288	0.123
Ctr.BMD (mg/mm^3^)	1486 ± 4.551	1485 ± 9.378	1488 ± 3.090	0.817
T.A (mm^2^)	9.7 ± 0.148	9.4 ± 0.248	9.5 ± 0.233	0.659
Ctr.A (mm^2^)	6.2 ± 0.134	5.7 ± 0.128	5.8 ± 0.201	0.075
Ctr.Th (mm)	0.7 ± 0.017	0.6 ± 0.011	0.7 ± 0.017	0.058
Peri.C (mm)	11.0 ± 0.085	10.8 ± 0.144	10.9 ± 0.132	0.650
Endo.C (mm)	6.5 ± 0.152	6.8 ± 0.131	6.8 ± 0.113	0.263
SSI (mm^3^)	7.5 ± 0.271	6.7 ± 0.306	7.1 ± 0.301	0.214

Data are expressed as means ± S.E.M. (*n* = 7 in each group); ^a,b,c^—means within a row with unlike superscripts differ significantly. Statistically significant differences were established at *p* ≤ 0.05. Abbrevations: T.BMC—total bone mineral content; Tv.BMD—total volumetric mineral density; Tb.BMC—trabecular bone mineral content; Tb.BMD—trabecular bone mineral density; T.A—total area; Tb.A—trabecular area; T.BMD—total mineral density; Ctr.BMC—cortical bone mineral content; Ctr.BMD—cortical bone mineral density; Ctr.A—cortical area; Ctr.Th—cortical thickness; Peri.C—peripheral circumference; Endo.C—endocortical circumference, SSI—strength strain index.

**Table 3:** Tibia tomographic parameters of female rats after the establishment of osteopenia.

Parameters	SHAM	OVA	NES	*p*-Value
Proximal metaphysis				
T.BMC (mg/mm)	14.1 ± 0.606 ^a^	10.0 ± 0.280 ^b^	10.6 ± 0.348 ^b^	<0.001
Tv.BMD (mg/mm^3^)	844 ± 13.36 ^a^	640 ± 20.22 ^c^	696 ± 23.30 ^b^	<0.001
Tb.BMC (mg/mm)	4.1 ± 0.276 ^a^	1.2 ± 0.056 ^b^	1.9 ± 0.089 ^ab^	<0.001
Tb.BMD (mg/mm^3^)	539 ± 22.4 ^a^	173 ± 12.05 ^c^	270 ± 16.64 ^b^	<0.001
T.A (mm^2^)	16.8 ± 0.728	15.7 ± 0.458	15.3 ± 0.776	0.319
Tb.A (mm^2^)	7.5 ± 0.325	7.1 ± 0.205	6.9 ± 0.348	0.327
Diaphysis				
T.BMC (mg/mm)	6.38 ± 0.193 ^a^	5.71 ± 0.091 ^b^	5.92 ± 0.083 ^b^	0.009
T.BMD (mg/mm^3^)	1122 ± 5.33	1113 ± 14.07	1145 ± 11.27	0.104
Ctr.BMC (mg/mm)	6.06 ± 0.198 ^a^	5.14± 0.206 ^b^	5.45 ± 0.072 ^ab^	0.006
Ctr.BMD (mg/mm^3^)	1449 ± 3.96 ^a^	1368 ± 7.183 ^b^	1439 ± 1.802 ^a^	0.049
T.A (mm^2^)	5.68 ± 0.164 ^a^	5.17 ± 0.081 ^b^	5.12 ± 0.055 ^b^	0.002
Ctr.A (mm^2^)	4.18 ± 0.144 ^a^	3.76 ± 0.048 ^b^	3.81 ± 0.048 ^b^	0.012
Ctr.Th (mm)	0.65 ± 0.015	0.62 ± 0.011	0.61 ± 0.012	0.219
Peri.C (mm)	8.44 ± 0.121 ^a^	8.03 ± 0.040 ^b^	8.05 ± 0.029 ^b^	0.002
Endo.C (mm)	4.35 ± 0.032	4.14 ± 0.107	4.18 ± 0.061	0.077
SSI (mm^3^)	3.49 ± 0.165 ^a^	2.80 ± 0.052 ^b^	3.01 ± 0.033 ^b^	0.002

Data are expressed as means ± S.E.M. (*n* = 7 in each group); ^a,b,c^—means within row with unlike superscripts differ significantly. Statistically significant differences were established at *p* ≤ 0.05. Abbrevations: such as Table 2.

**Table 4:** Strength parameters of isolated bones of female rats after the establishment of osteopenia.

Parameters	SHAM	OVA	NES	*p*-Value
Femur				
Young’s modulus (GPa)	4.59 ± 0.568	4.56 ± 0.295	4.63 ± 0.222	0.993
Ultimate strength (N)	135 ± 4.010 ^a^	109 ± 7.037 ^b^	140 ± 9.257 ^a^	0.013
Work to fracture (mJ)	27.86 ± 1.447 ^a^	19.55 ± 1.714 ^b^	25.38 ± 1.876 ^a^	0.007
Tibia				
Young’s modulus (GPa)	3.67 ± 0.205 ^a^	5.44 ± 0.494 ^ab^	6.05 ± 1.003 ^b^	0.050
Ultimate strength (N)	93.45 ± 5.27	83.45 ± 2.76	97 ± 5.02	0.081
Work to fracture (mJ)	20.13 ± 2.058	18.78 ± 1.355	21.48 ± 2.07	0.729

Data are expressed as means ± S.E.M. (*n* = 7 in each group); ^a,b^—means within a row with unlike superscripts differ significantly. Statistically significant differences were established at *p* ≤ 0.05.

**Table 5:** Serum concentration of bone metabolism markers, ionized calcium and phosphorus of female rats after the establishment of osteopenia.

Parameters	SHAM	OVA	NES	*p*-Value
Osteocalcin (ng/mL)	4.62 ± 0.310	4.87 ± 0.240	4.41 ± 0.452	0.650
bALP (ng/mL)	15.422 ± 0.793 ^ab^	14.356 ± 0.837 ^a^	17.281 ± 0.306 ^b^	0.039
NTx (nmol/mL)	23.051 ± 0.624 ^a^	25.376 ± 0.536 ^b^	24.046 ± 0.659 ^ab^	0.044
Ca (mg/dL)	9.56 ± 0.14	10.44 ± 0.49	10.46 ± 0.98	0.156
P (mg/dL)	8.68 ± 0.42	8.33 ± 1.21	10.67 ± 0.67	0.128

Data are expressed as means ± S.E.M. (*n* = 7 in each group); ^a,b^—means within a row with unlike superscripts differ significantly. Statistically significant differences were established at *p* ≤ 0.05. Abbreviations: bALP—bone specific alkaline phosphatase; NTx—N-terminal crosslinked telopeptide type I collagen; Ca—calcium; P—phosphorus.

## Data Availability

The data presented in this study are available on request from the corresponding author.
